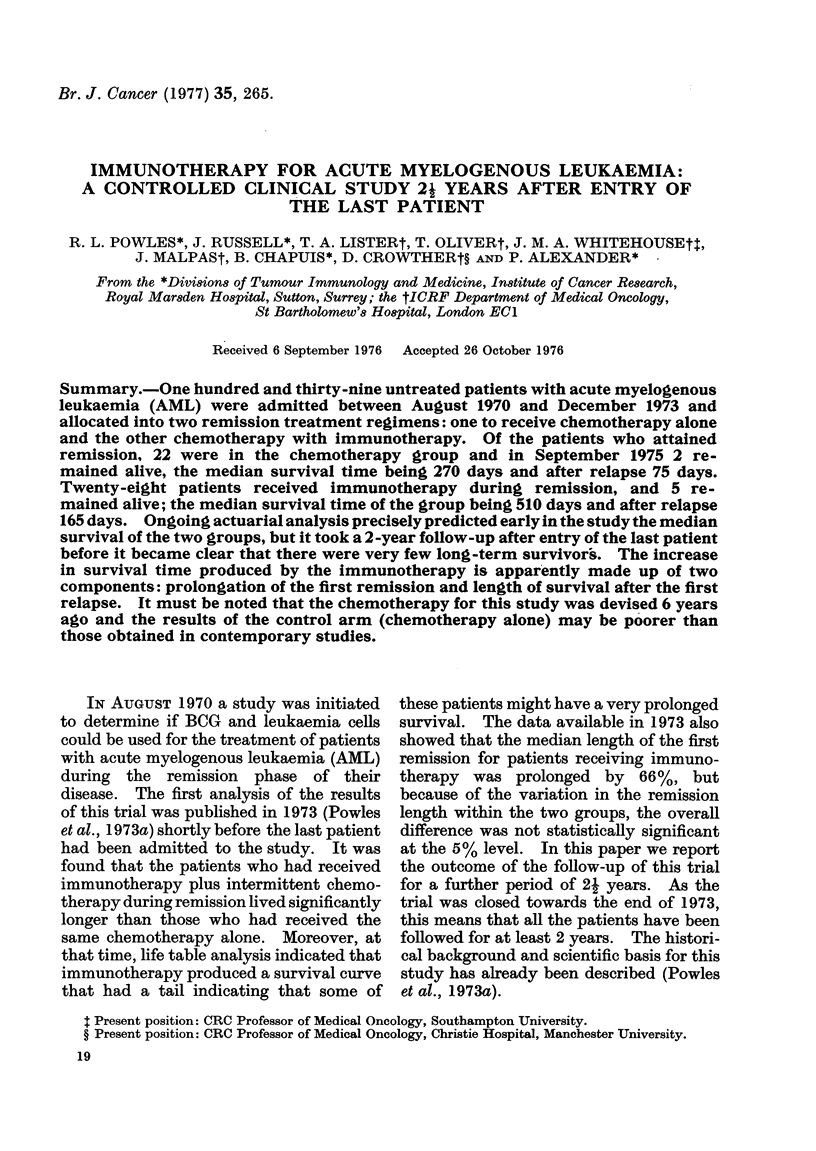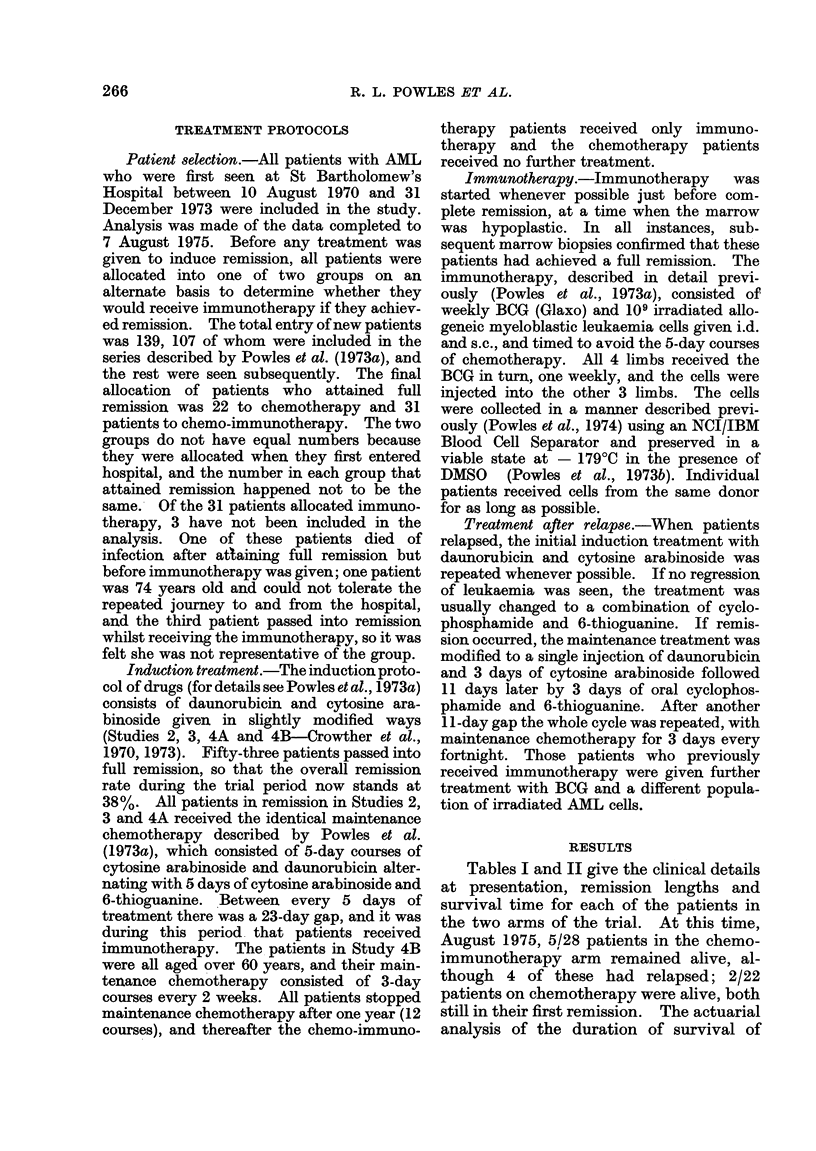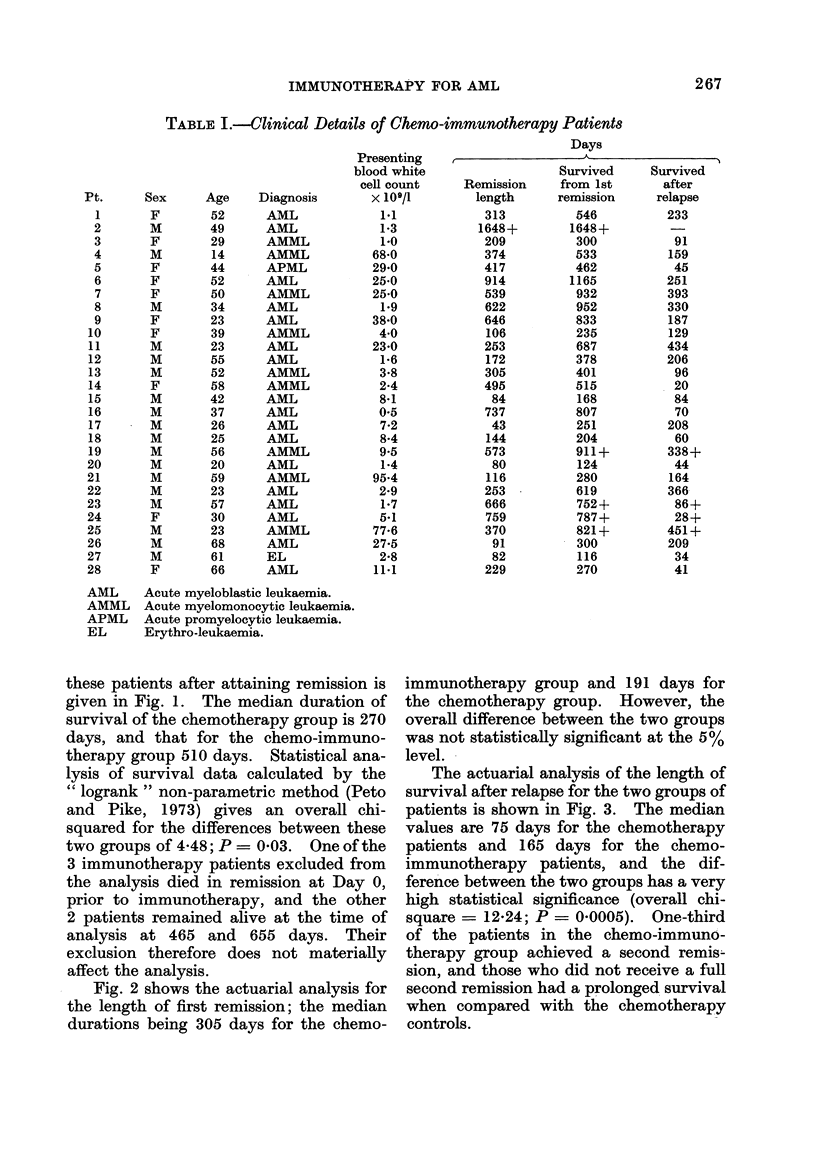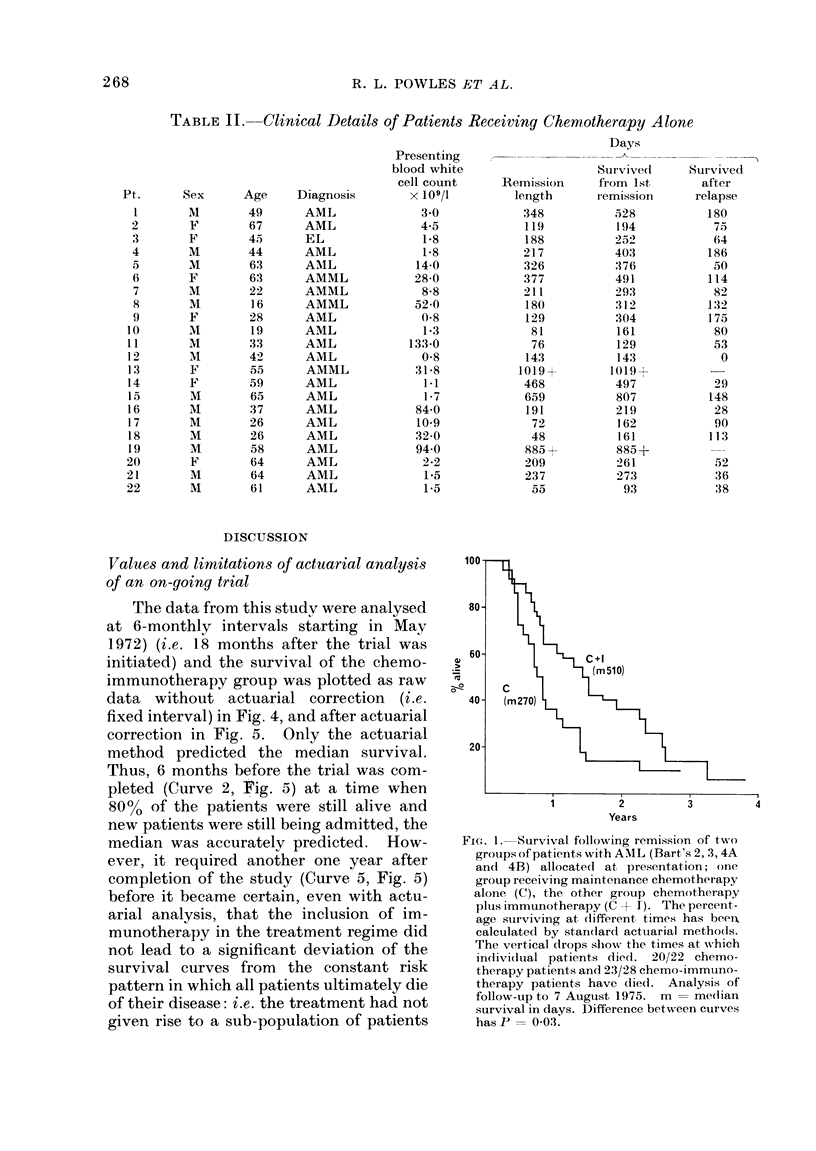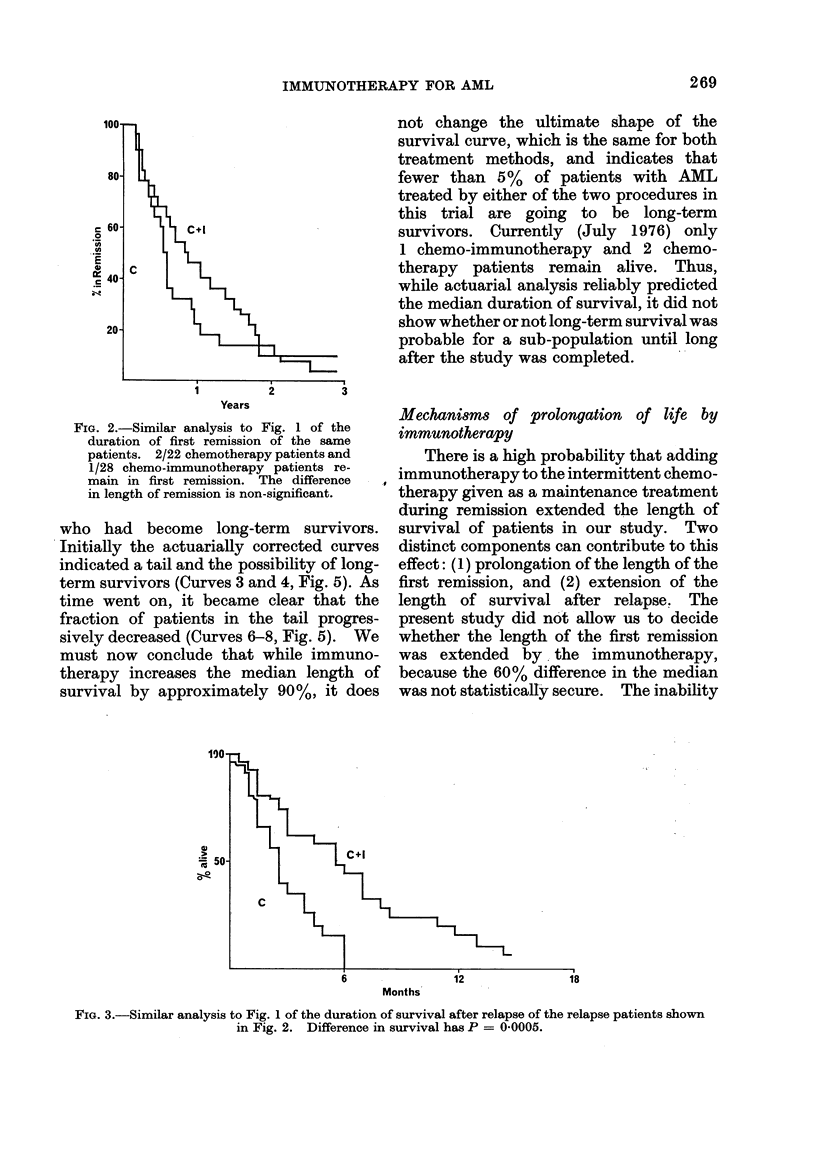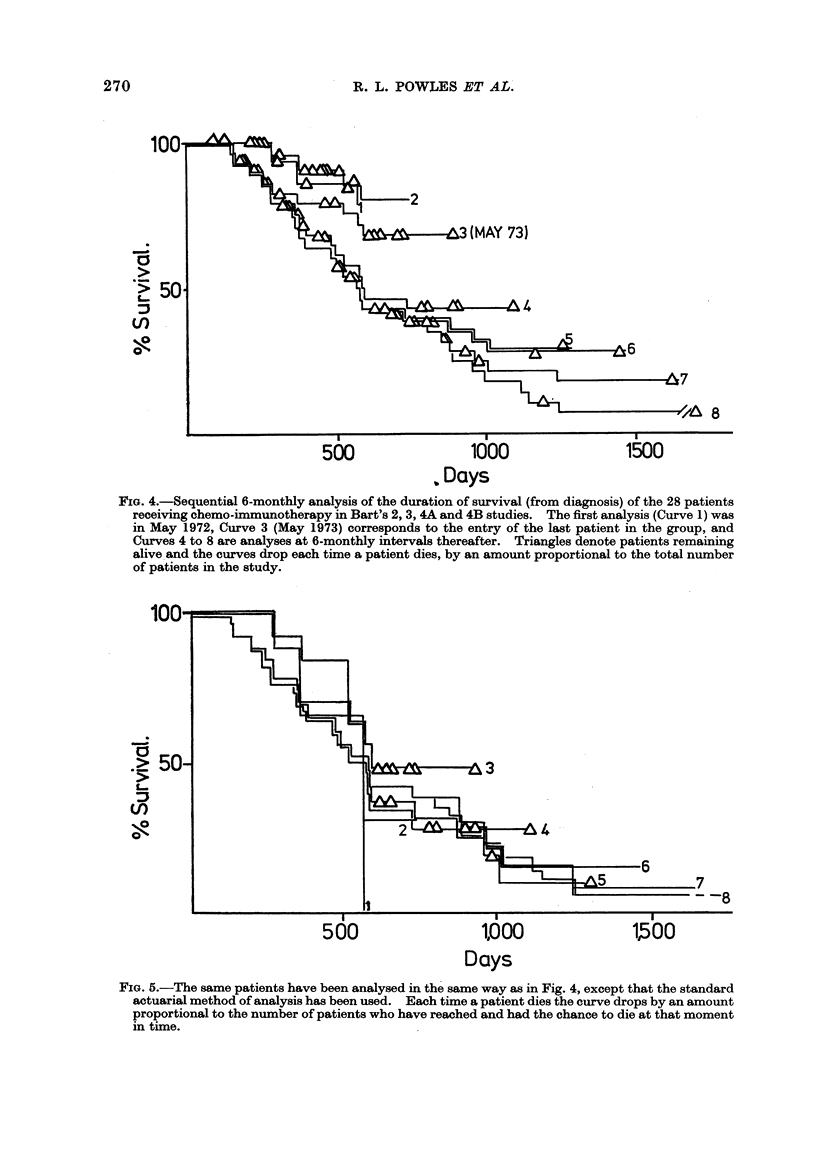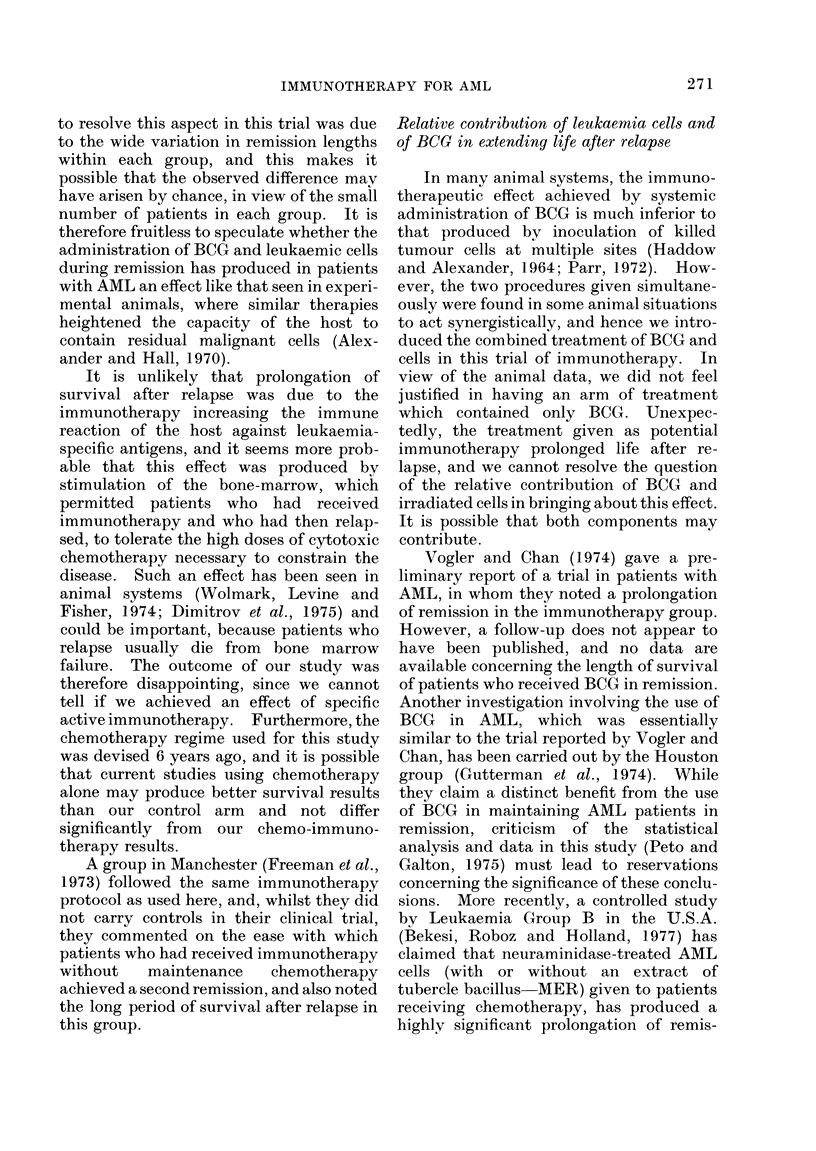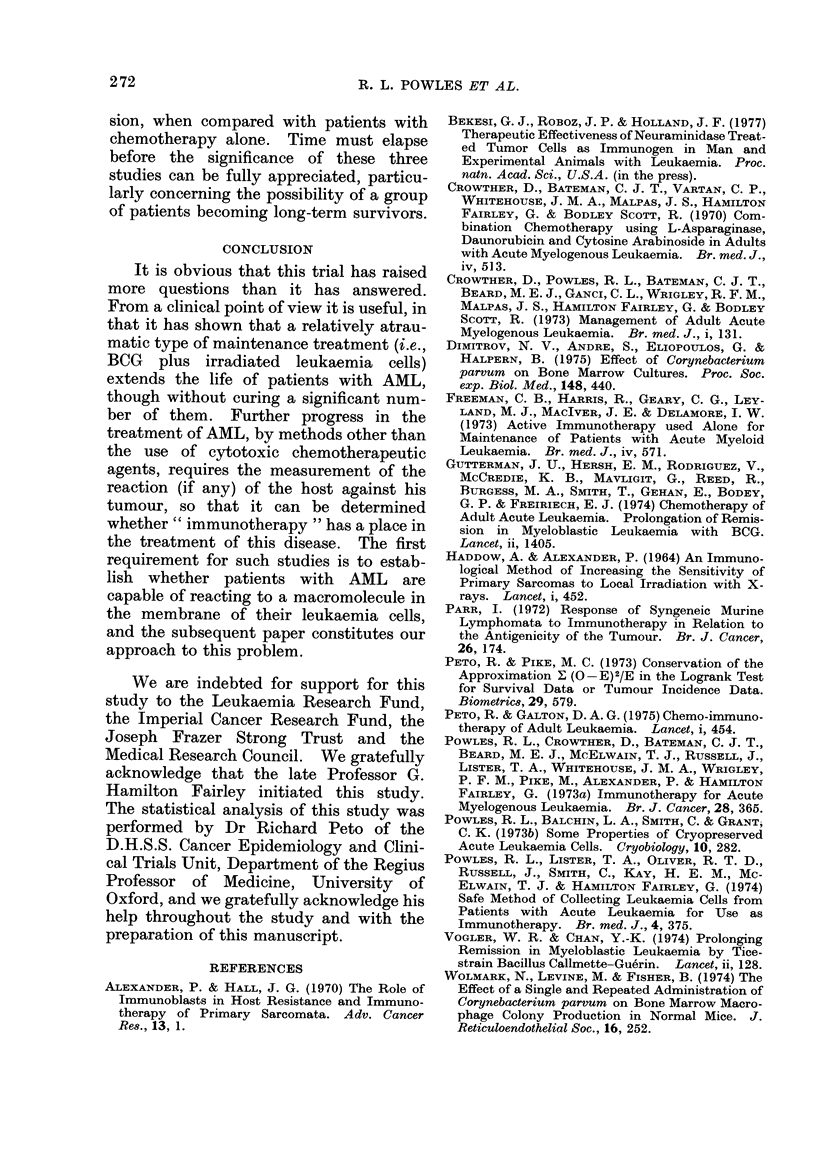# Immunotherapy for acute myelogenous leukaemia: a controlled clinical study 2 1/2 years after entry of the last patient.

**DOI:** 10.1038/bjc.1977.38

**Published:** 1977-03

**Authors:** R. L. Powles, J. Russell, T. A. Lister, T. Oliver, J. M. Whitehouse, J. Malpas, B. Chapuis, D. Crowther, P. Alexander

## Abstract

One hundred and thirty-nine untreated patients with acute myelogenous leukaemia (AML) were admitted between August 1970 and December 1973 and allocated into two remission treatment regimens: one to receive chemotherapy alone and the other chemotherapy with immunotherapy. Of the patients who attained remission. 22 were in the chemotherapy group and in September 1975 2 remained alive, the median survival time being 270 days and after relapse 75 days. Twenty-eight patients received immunotherapy during remission, and 5 remained alive; the median survival time of the group being 510 days and after relapse 165 days. Ongoing acturial analysis precisely predicted early in the study the median survival of the two groups, but it took a 2-year follow-up after entry of the last patient before it became clear that there were very few long-term survivors. The increase in survival time produced by the immunotherapy is apparently made up of two components: prolongation of the first remission and length of survival after the first relapse. It must be notted that the chemotherapy for this study was devised 6 years ago and the results of the control arm (chemotherapy alone) may be poorer than those obtained in contemporary studies.


*Br. J. Cancer* (1977) **35**, 265.

### IMMUNOTHERAPY FOR ACUTE MYELOGENOUS LEUKAEMIA: A CONTROLLED CLINICAL STUDY 2½ YEARS AFTER ENTRY OF THE LAST PATIENT

R. L. POWLES*, J. RUSSELL*, T. A. LISTER†, T. OLIVER†, J. M. A. WHITEHOUSE†‡, J. MALPAS†, B. CHAPUIS*, D. CROWTHER†§ AND P. ALEXANDER*

*From the *Divisions of Tumour Immunology and Medicine, Institute of Cancer Research, Royal Marsden Hospital, Sutton, Surrey; the †ICRF Department of Medical Oncology, St Bartholomew's Hospital, London EC1*

Received 6 September 1976 Accepted 26 October 1976

**Summary.—One hundred and thirty-nine untreated patients with acute myelogenous leukaemia (AML) were admitted between August 1970 and December 1973 and allocated into two remission treatment regimens: one to receive chemotherapy alone and the other chemotherapy with immunotherapy. Of the patients who attained remission, 22 were in the chemotherapy group and in September 1975 2 remained alive, the median survival time being 270 days and after relapse 75 days. Twenty-eight patients received immunotherapy during remission, and 5 remained alive; the median survival time of the group being 510 days and after relapse 165 days. Ongoing actuarial analysis precisely predicted early in the study the median survival of the two groups, but it took a 2-year follow-up after entry of the last patient before it became clear that there were very few long-term survivors. The increase in survival time produced by the immunotherapy is apparently made up of two components: prolongation of the first remission and length of survival after the first relapse. It must be noted that the chemotherapy for this study was devised 6 years ago and the results of the control arm (chemotherapy alone) may be poorer than those obtained in contemporary studies.**

IN AUGUST 1970 a study was initiated to determine if BCG and leukaemia cells could be used for the treatment of patients with acute myelogenous leukaemia (AML) during the remission phase of their disease. The first analysis of the results of this trial was published in 1973 (Powles *et al.*, 1973*a*) shortly before the last patient had been admitted to the study. It was found that the patients who had received immunotherapy plus intermittent chemotherapy during remission lived significantly longer than those who had received the same chemotherapy alone. Moreover, at that time, life table analysis indicated that immunotherapy produced a survival curve that had a tail indicating that some of these patients might have a very prolonged survival. The data available in 1973 also showed that the median length of the first remission for patients receiving immunotherapy was prolonged by 66%, but because of the variation in the remission length within the two groups, the overall difference was not statistically significant at the 5% level. In this paper we report the outcome of the follow-up of this trial for a further period of 2½ years. As the trial was closed towards the end of 1973, this means that all the patients have been followed for at least 2 years. The historical background and scientific basis for this study has already been described (Powles *et al.*, 1973*a*).

‡ Present position: CRC Professor of Medical Oncology, Southampton University.
§ Present position: CRC Professor of Medical Oncology, Christie Hospital, Manchester University.

### TREATMENT PROTOCOLS

*Patient selection.*—All patients with AML who were first seen at St Bartholomew's Hospital between 10 August 1970 and 31 December 1973 were included in the study. Analysis was made of the data completed to 7 August 1975. Before any treatment was given to induce remission, all patients were allocated into one of two groups on an alternate basis to determine whether they would receive immunotherapy if they achieved remission. The total entry of new patients was 139, 107 of whom were included in the series described by Powles *et al.* (1973*a*), and the rest were seen subsequently. The final allocation of patients who attained full remission was 22 to chemotherapy and 31 patients to chemo-immunotherapy. The two groups do not have equal numbers because they were allocated when they first entered hospital, and the number in each group that attained remission happened not to be the same. Of the 31 patients allocated immunotherapy, 3 have not been included in the analysis. One of these patients died of infection after attaining full remission but before immunotherapy was given; one patient was 74 years old and could not tolerate the repeated journey to and from the hospital, and the third patient passed into remission whilst receiving the immunotherapy, so it was felt she was not representative of the group.

*Induction treatment.*—The induction protocol of drugs (for details see Powles *et al.*, 1973*a*) consists of daunorubicin and cytosine arabinoside given in slightly modified ways (Studies 2, 3, 4A and 4B—Crowther *et al.*, 1970, 1973). Fifty-three patients passed into full remission, so that the overall remission rate during the trial period now stands at 38%. All patients in remission in Studies 2, 3 and 4A received the identical maintenance chemotherapy described by Powles *et al.* (1973*a*), which consisted of 5-day courses of cytosine arabinoside and daunorubicin alternating with 5 days of cytosine arabinoside and 6-thioguanine. Between every 5 days of treatment there was a 23-day gap, and it was during this period that patients received immunotherapy. The patients in Study 4B were all aged over 60 years, and their maintenance chemotherapy consisted of 3-day courses every 2 weeks. All patients stopped maintenance chemotherapy after one year (12 courses), and thereafter the chemo-immunotherapy patients received only immunotherapy and the chemotherapy patients received no further treatment.

*Immunotherapy.*—Immunotherapy was started whenever possible just before complete remission, at a time when the marrow was hypoplastic. In all instances, subsequent marrow biopsies confirmed that these patients had achieved a full remission. The immunotherapy, described in detail previously (Powles *et al.*, 1973*a*), consisted of weekly BCG (Glaxo) and $10^9$ irradiated allogeneic myeloblastic leukaemia cells given i.d. and s.c., and timed to avoid the 5-day courses of chemotherapy. All 4 limbs received the BCG in turn, one weekly, and the cells were injected into the other 3 limbs. The cells were collected in a manner described previously (Powles *et al.*, 1974) using an NCI/IBM Blood Cell Separator and preserved in a viable state at − 179°C in the presence of DMSO (Powles *et al.*, 1973*b*). Individual patients received cells from the same donor for as long as possible.

*Treatment after relapse.*—When patients relapsed, the initial induction treatment with daunorubicin and cytosine arabinoside was repeated whenever possible. If no regression of leukaemia was seen, the treatment was usually changed to a combination of cyclophosphamide and 6-thioguanine. If remission occurred, the maintenance treatment was modified to a single injection of daunorubicin and 3 days of cytosine arabinoside followed 11 days later by 3 days of oral cyclophosphamide and 6-thioguanine. After another 11-day gap the whole cycle was repeated, with maintenance chemotherapy for 3 days every fortnight. Those patients who previously received immunotherapy were given further treatment with BCG and a different population of irradiated AML cells.

### RESULTS

Tables I and II give the clinical details at presentation, remission lengths and survival time for each of the patients in the two arms of the trial. At this time, August 1975, 5/28 patients in the chemo-immunotherapy arm remained alive, although 4 of these had relapsed; 2/22 patients on chemotherapy were alive, both still in their first remission. The actuarial analysis of the duration of survival of

TABLE I.—*Clinical Details of Chemo-immunotherapy Patients*

| Pt. | Sex | Age | Diagnosis | Presenting blood white cell count $\times 10^9$/l | Days: Remission length | Days: Survived from 1st remission | Days: Survived after relapse |
|---|---|---|---|---|---|---|---|
| 1 | F | 52 | AML | 1·1 | 313 | 546 | 233 |
| 2 | M | 49 | AML | 1·3 | 1648+ | 1648+ | — |
| 3 | F | 29 | AMML | 1·0 | 209 | 300 | 91 |
| 4 | M | 14 | AMML | 68·0 | 374 | 533 | 159 |
| 5 | F | 44 | APML | 29·0 | 417 | 462 | 45 |
| 6 | F | 52 | AML | 25·0 | 914 | 1165 | 251 |
| 7 | F | 50 | AMML | 25·0 | 539 | 932 | 393 |
| 8 | M | 34 | AML | 1·9 | 622 | 952 | 330 |
| 9 | F | 23 | AML | 38·0 | 646 | 833 | 187 |
| 10 | F | 39 | AMML | 4·0 | 106 | 235 | 129 |
| 11 | M | 23 | AML | 23·0 | 253 | 687 | 434 |
| 12 | M | 55 | AML | 1·6 | 172 | 378 | 206 |
| 13 | M | 52 | AMML | 3·8 | 305 | 401 | 96 |
| 14 | F | 58 | AMML | 2·4 | 495 | 515 | 20 |
| 15 | M | 42 | AML | 8·1 | 84 | 168 | 84 |
| 16 | M | 37 | AML | 0·5 | 737 | 807 | 70 |
| 17 | M | 26 | AML | 7·2 | 43 | 251 | 208 |
| 18 | M | 25 | AML | 8·4 | 144 | 204 | 60 |
| 19 | M | 56 | AMML | 9·5 | 573 | 911+ | 338+ |
| 20 | M | 20 | AML | 1·4 | 80 | 124 | 44 |
| 21 | M | 59 | AMML | 95·4 | 116 | 280 | 164 |
| 22 | M | 23 | AML | 2·9 | 253 | 619 | 366 |
| 23 | M | 57 | AML | 1·7 | 666 | 752+ | 86+ |
| 24 | F | 30 | AML | 5·1 | 759 | 787+ | 28+ |
| 25 | M | 23 | AMML | 77·6 | 370 | 821+ | 451+ |
| 26 | M | 68 | AML | 27·5 | 91 | 300 | 209 |
| 27 | M | 61 | EL | 2·8 | 82 | 116 | 34 |
| 28 | F | 66 | AML | 11·1 | 229 | 270 | 41 |

AML Acute myeloblastic leukaemia.

AMML Acute myelomonocytic leukaemia.

APML Acute promyelocytic leukaemia.

EL Erythro-leukaemia.

these patients after attaining remission is given in Fig. 1. The median duration of survival of the chemotherapy group is 270 days, and that for the chemo-immunotherapy group 510 days. Statistical analysis of survival data calculated by the " logrank " non-parametric method (Peto and Pike, 1973) gives an overall chi-squared for the differences between these two groups of 4·48; $P = 0{\cdot}03$. One of the 3 immunotherapy patients excluded from the analysis died in remission at Day 0, prior to immunotherapy, and the other 2 patients remained alive at the time of analysis at 465 and 655 days. Their exclusion therefore does not materially affect the analysis.

Fig. 2 shows the actuarial analysis for the length of first remission; the median durations being 305 days for the chemo-immunotherapy group and 191 days for the chemotherapy group. However, the overall difference between the two groups was not statistically significant at the 5% level.

The actuarial analysis of the length of survival after relapse for the two groups of patients is shown in Fig. 3. The median values are 75 days for the chemotherapy patients and 165 days for the chemo-immunotherapy patients, and the difference between the two groups has a very high statistical significance (overall chi-square = 12·24; $P = 0{\cdot}0005$). One-third of the patients in the chemo-immunotherapy group achieved a second remission, and those who did not receive a full second remission had a prolonged survival when compared with the chemotherapy controls.

TABLE II.—*Clinical Details of Patients Receiving Chemotherapy Alone*

| Pt. | Sex | Age | Diagnosis | Presenting blood white cell count $\times 10^9$/l | Days: Remission length | Days: Survived from 1st remission | Days: Survived after relapse |
|---|---|---|---|---|---|---|---|
| 1 | M | 49 | AML | 3·0 | 348 | 528 | 180 |
| 2 | F | 67 | AML | 4·5 | 119 | 194 | 75 |
| 3 | F | 45 | EL | 1·8 | 188 | 252 | 64 |
| 4 | M | 44 | AML | 1·8 | 217 | 403 | 186 |
| 5 | M | 63 | AML | 14·0 | 326 | 376 | 50 |
| 6 | F | 63 | AMML | 28·0 | 377 | 491 | 114 |
| 7 | M | 22 | AMML | 8·8 | 211 | 293 | 82 |
| 8 | M | 16 | AMML | 52·0 | 180 | 312 | 132 |
| 9 | F | 28 | AML | 0·8 | 129 | 304 | 175 |
| 10 | M | 19 | AML | 1·3 | 81 | 161 | 80 |
| 11 | M | 33 | AML | 133·0 | 76 | 129 | 53 |
| 12 | M | 42 | AML | 0·8 | 143 | 143 | 0 |
| 13 | F | 55 | AMML | 31·8 | 1019+ | 1019+ | — |
| 14 | F | 59 | AML | 1·1 | 468 | 497 | 29 |
| 15 | M | 65 | AML | 1·7 | 659 | 807 | 148 |
| 16 | M | 37 | AML | 84·0 | 191 | 219 | 28 |
| 17 | M | 26 | AML | 10·9 | 72 | 162 | 90 |
| 18 | M | 26 | AML | 32·0 | 48 | 161 | 113 |
| 19 | M | 58 | AML | 94·0 | 885+ | 885+ | — |
| 20 | F | 64 | AML | 2·2 | 209 | 261 | 52 |
| 21 | M | 64 | AML | 1·5 | 237 | 273 | 36 |
| 22 | M | 61 | AML | 1·5 | 55 | 93 | 38 |

### DISCUSSION

### *Values and limitations of actuarial analysis of an on-going trial*

The data from this study were analysed at 6-monthly intervals starting in May 1972) (*i.e.* 18 months after the trial was initiated) and the survival of the chemo-immunotherapy group was plotted as raw data without actuarial correction (*i.e.* fixed interval) in Fig. 4, and after actuarial correction in Fig. 5. Only the actuarial method predicted the median survival. Thus, 6 months before the trial was completed (Curve 2, Fig. 5) at a time when 80% of the patients were still alive and new patients were still being admitted, the median was accurately predicted. However, it required another one year after completion of the study (Curve 5, Fig. 5) before it became certain, even with actuarial analysis, that the inclusion of immunotherapy in the treatment regime did not lead to a significant deviation of the survival curves from the constant risk pattern in which all patients ultimately die of their disease: *i.e.* the treatment had not given rise to a sub-population of patients

FIG. 1.—Survival following remission of two groups of patients with AML (Bart's 2, 3, 4A and 4B) allocated at presentation; one group receiving maintenance chemotherapy alone (C), the other group chemotherapy plus immunotherapy (C + I). The percentage surviving at different times has been calculated by standard actuarial methods. The vertical drops show the times at which individual patients died. 20/22 chemotherapy patients and 23/28 chemo-immunotherapy patients have died. Analysis of follow-up to 7 August 1975. m = median survival in days. Difference between curves has $P = 0{\cdot}03$.

FIG. 2.—Similar analysis to Fig. 1 of the duration of first remission of the same patients. 2/22 chemotherapy patients and 1/28 chemo-immunotherapy patients remain in first remission. The difference in length of remission is non-significant.

who had become long-term survivors. Initially the actuarially corrected curves indicated a tail and the possibility of long-term survivors (Curves 3 and 4, Fig. 5). As time went on, it became clear that the fraction of patients in the tail progressively decreased (Curves 6–8, Fig. 5). We must now conclude that while immunotherapy increases the median length of survival by approximately 90%, it does not change the ultimate shape of the survival curve, which is the same for both treatment methods, and indicates that fewer than 5% of patients with AML treated by either of the two procedures in this trial are going to be long-term survivors. Currently (July 1976) only 1 chemo-immunotherapy and 2 chemotherapy patients remain alive. Thus, while actuarial analysis reliably predicted the median duration of survival, it did not show whether or not long-term survival was probable for a sub-population until long after the study was completed.

*Mechanisms of prolongation of life by immunotherapy*

There is a high probability that adding immunotherapy to the intermittent chemotherapy given as a maintenance treatment during remission extended the length of survival of patients in our study. Two distinct components can contribute to this effect: (1) prolongation of the length of the first remission, and (2) extension of the length of survival after relapse. The present study did not allow us to decide whether the length of the first remission was extended by the immunotherapy, because the 60% difference in the median was not statistically secure. The inability

FIG. 3.—Similar analysis to Fig. 1 of the duration of survival after relapse of the relapse patients shown in Fig. 2. Difference in survival has $P = 0{\cdot}0005$.

FIG. 4.—Sequential 6-monthly analysis of the duration of survival (from diagnosis) of the 28 patients receiving chemo-immunotherapy in Bart's 2, 3, 4A and 4B studies. The first analysis (Curve 1) was in May 1972, Curve 3 (May 1973) corresponds to the entry of the last patient in the group, and Curves 4 to 8 are analyses at 6-monthly intervals thereafter. Triangles denote patients remaining alive and the curves drop each time a patient dies, by an amount proportional to the total number of patients in the study.

FIG. 5.—The same patients have been analysed in the same way as in Fig. 4, except that the standard actuarial method of analysis has been used. Each time a patient dies the curve drops by an amount proportional to the number of patients who have reached and had the chance to die at that moment in time.

to resolve this aspect in this trial was due to the wide variation in remission lengths within each group, and this makes it possible that the observed difference may have arisen by chance, in view of the small number of patients in each group. It is therefore fruitless to speculate whether the administration of BCG and leukaemic cells during remission has produced in patients with AML an effect like that seen in experimental animals, where similar therapies heightened the capacity of the host to contain residual malignant cells (Alexander and Hall, 1970).

It is unlikely that prolongation of survival after relapse was due to the immunotherapy increasing the immune reaction of the host against leukaemia-specific antigens, and it seems more probable that this effect was produced by stimulation of the bone-marrow, which permitted patients who had received immunotherapy and who had then relapsed, to tolerate the high doses of cytotoxic chemotherapy necessary to constrain the disease. Such an effect has been seen in animal systems (Wolmark, Levine and Fisher, 1974; Dimitrov *et al.*, 1975) and could be important, because patients who relapse usually die from bone marrow failure. The outcome of our study was therefore disappointing, since we cannot tell if we achieved an effect of specific active immunotherapy. Furthermore, the chemotherapy regime used for this study was devised 6 years ago, and it is possible that current studies using chemotherapy alone may produce better survival results than our control arm and not differ significantly from our chemo-immunotherapy results.

A group in Manchester (Freeman *et al.*, 1973) followed the same immunotherapy protocol as used here, and, whilst they did not carry controls in their clinical trial, they commented on the ease with which patients who had received immunotherapy without maintenance chemotherapy achieved a second remission, and also noted the long period of survival after relapse in this group.

*Relative contribution of leukaemia cells and of BCG in extending life after relapse*

In many animal systems, the immunotherapeutic effect achieved by systemic administration of BCG is much inferior to that produced by inoculation of killed tumour cells at multiple sites (Haddow and Alexander, 1964; Parr, 1972). However, the two procedures given simultaneously were found in some animal situations to act synergistically, and hence we introduced the combined treatment of BCG and cells in this trial of immunotherapy. In view of the animal data, we did not feel justified in having an arm of treatment which contained only BCG. Unexpectedly, the treatment given as potential immunotherapy prolonged life after relapse, and we cannot resolve the question of the relative contribution of BCG and irradiated cells in bringing about this effect. It is possible that both components may contribute.

Vogler and Chan (1974) gave a preliminary report of a trial in patients with AML, in whom they noted a prolongation of remission in the immunotherapy group. However, a follow-up does not appear to have been published, and no data are available concerning the length of survival of patients who received BCG in remission. Another investigation involving the use of BCG in AML, which was essentially similar to the trial reported by Vogler and Chan, has been carried out by the Houston group (Gutterman *et al.*, 1974). While they claim a distinct benefit from the use of BCG in maintaining AML patients in remission, criticism of the statistical analysis and data in this study (Peto and Galton, 1975) must lead to reservations concerning the significance of these conclusions. More recently, a controlled study by Leukaemia Group B in the U.S.A. (Bekesi, Roboz and Holland, 1977) has claimed that neuraminidase-treated AML cells (with or without an extract of tubercle bacillus—MER) given to patients receiving chemotherapy, has produced a highly significant prolongation of remission, when compared with patients with chemotherapy alone. Time must elapse before the significance of these three studies can be fully appreciated, particularly concerning the possibility of a group of patients becoming long-term survivors.

### CONCLUSION

It is obvious that this trial has raised more questions than it has answered. From a clinical point of view it is useful, in that it has shown that a relatively atraumatic type of maintenance treatment (*i.e.*, BCG plus irradiated leukaemia cells) extends the life of patients with AML, though without curing a significant number of them. Further progress in the treatment of AML, by methods other than the use of cytotoxic chemotherapeutic agents, requires the measurement of the reaction (if any) of the host against his tumour, so that it can be determined whether " immunotherapy " has a place in the treatment of this disease. The first requirement for such studies is to establish whether patients with AML are capable of reacting to a macromolecule in the membrane of their leukaemia cells, and the subsequent paper constitutes our approach to this problem.

We are indebted for support for this study to the Leukaemia Research Fund, the Imperial Cancer Research Fund, the Joseph Frazer Strong Trust and the Medical Research Council. We gratefully acknowledge that the late Professor G. Hamilton Fairley initiated this study. The statistical analysis of this study was performed by Dr Richard Peto of the D.H.S.S. Cancer Epidemiology and Clinical Trials Unit, Department of the Regius Professor of Medicine, University of Oxford, and we gratefully acknowledge his help throughout the study and with the preparation of this manuscript.

### REFERENCES

ALEXANDER, P. & HALL, J. G. (1970) The Role of Immunoblasts in Host Resistance and Immunotherapy of Primary Sarcomata. *Adv. Cancer Res.*, **13**, 1.

BEKESI, G. J., ROBOZ, J. P. & HOLLAND, J. F. (1977) Therapeutic Effectiveness of Neuraminidase Treated Tumor Cells as Immunogen in Man and Experimental Animals with Leukaemia. *Proc. natn. Acad. Sci., U.S.A.* (in the press).

CROWTHER, D., BATEMAN, C. J. T., VARTAN, C. P., WHITEHOUSE, J. M. A., MALPAS, J. S., HAMILTON FAIRLEY, G. & BODLEY SCOTT, R. (1970) Combination Chemotherapy using L-Asparaginase, Daunorubicin and Cytosine Arabinoside in Adults with Acute Myelogenous Leukaemia. *Br. med. J.*, iv, 513.

CROWTHER, D., POWLES, R. L., BATEMAN, C. J. T., BEARD, M. E. J., GANCI, C. L., WRIGLEY, R. F. M., MALPAS, J. S., HAMILTON FAIRLEY, G. & BODLEY SCOTT, R. (1973) Management of Adult Acute Myelogenous Leukaemia. *Br. med. J.*, i, 131.

DIMITROV, N. V., ANDRE, S., ELIOPOULOS, G. & HALPERN, B. (1975) Effect of *Corynebacterium parvum* on Bone Marrow Cultures. *Proc. Soc. exp. Biol. Med.*, **148**, 440.

FREEMAN, C. B., HARRIS, R., GEARY, C. G., LEYLAND, M. J., MACIVER, J. E. & DELAMORE, I. W. (1973) Active Immunotherapy used Alone for Maintenance of Patients with Acute Myeloid Leukaemia. *Br. med. J.*, iv, 571.

GUTTERMAN, J. U., HERSH, E. M., RODRIGUEZ, V., MCCREDIE, K. B., MAVLIGIT, G., REED, R., BURGESS, M. A., SMITH, T., GEHAN, E., BODEY, G. P. & FREIRIECH, E. J. (1974) Chemotherapy of Adult Acute Leukaemia. Prolongation of Remission in Myeloblastic Leukaemia with BCG. *Lancet*, ii, 1405.

HADDOW, A. & ALEXANDER, P. (1964) An Immunological Method of Increasing the Sensitivity of Primary Sarcomas to Local Irradiation with X-rays. *Lancet*, i, 452.

PARR, I. (1972) Response of Syngeneic Murine Lymphomata to Immunotherapy in Relation to the Antigenicity of the Tumour. *Br. J. Cancer*, **26**, 174.

PETO, R. & PIKE, M. C. (1973) Conservation of the Approximation $\Sigma\,(O-E)^2/E$ in the Logrank Test for Survival Data or Tumour Incidence Data. *Biometrics*, **29**, 579.

PETO, R. & GALTON, D. A. G. (1975) Chemo-immunotherapy of Adult Leukaemia. *Lancet*, i, 454.

POWLES, R. L., CROWTHER, D., BATEMAN, C. J. T., BEARD, M. E. J., MCELWAIN, T. J., RUSSELL, J., LISTER, T. A., WHITEHOUSE, J. M. A., WRIGLEY, P. F. M., PIKE, M., ALEXANDER, P. & HAMILTON FAIRLEY, G. (1973*a*) Immunotherapy for Acute Myelogenous Leukaemia. *Br. J. Cancer*, **28**, 365.

POWLES, R. L., BALCHIN, L. A., SMITH, C. & GRANT, C. K. (1973*b*) Some Properties of Cryopreserved Acute Leukaemia Cells. *Cryobiology*, **10**, 282.

POWLES, R. L., LISTER, T. A., OLIVER, R. T. D., RUSSELL, J., SMITH, C., KAY, H. E. M., MCELWAIN, T. J. & HAMILTON FAIRLEY, G. (1974) Safe Method of Collecting Leukaemia Cells from Patients with Acute Leukaemia for Use as Immunotherapy. *Br. med. J.*, **4**, 375.

VOGLER, W. R. & CHAN, Y.-K. (1974) Prolonging Remission in Myeloblastic Leukaemia by Tice-strain Bacillus Callmette–Guérin. *Lancet*, ii, 128.

WOLMARK, N., LEVINE, M. & FISHER, B. (1974) The Effect of a Single and Repeated Administration of *Corynebacterium parvum* on Bone Marrow Macrophage Colony Production in Normal Mice. *J. Reticuloendothelial Soc.*, **16**, 252.